# The Multiple Sclerosis Risk Sharing Scheme Monitoring Study – early results and lessons for the future

**DOI:** 10.1186/1471-2377-9-1

**Published:** 2009-01-06

**Authors:** Mark Pickin, Cindy L Cooper, Timothy Chater, Anthony O'Hagan, Keith R Abrams, Nicola J Cooper, Mike Boggild, Jackie Palace, George Ebers, James B Chilcott, Paul Tappenden, Jon Nicholl

**Affiliations:** 1 Medical Care Research Unit, School of Health and Related Research, University of Sheffield, Regent Court, 30 Regent Street, Sheffield, S1 4DA, UK; 2 Department of Probability and Statistics, University of Sheffield, The Hicks Building, Hounsfield Road, Sheffield, S3 7RH, UK; 3 Department of Health Sciences, University of Leicester, Adrian Building, University Road, Leicester, LE1 7RH, UK; 4 The Walton Centre, Liverpool, L9 7LJ, UK; 5 University of Oxford Department of Clinical Neurology, Level 3, West Wing, John Radcliffe Hospital, Oxford, OX3 9DU, UK; 6 Health Economics and Decision Science Section, School of Health and Related Research, University of Sheffield, Regent Court, 30 Regent Street, Sheffield, S1 4DA, UK

## Abstract

**Background:**

Risk sharing schemes represent an innovative and important approach to the problems of rationing and achieving cost-effectiveness in high cost or controversial health interventions. This study aimed to assess the feasibility of risk sharing schemes, looking at long term clinical outcomes, to determine the price at which high cost treatments would be acceptable to the NHS.

**Methods:**

This case study of the first NHS risk sharing scheme, a long term prospective cohort study of beta interferon and glatiramer acetate in multiple sclerosis (MS) patients in 71 specialist MS centres in UK NHS hospitals, recruited adults with relapsing forms of MS, meeting Association of British Neurologists (ABN) criteria for disease modifying therapy. Outcome measures were: success of recruitment and follow up over the first three years, analysis of baseline and initial follow up data and the prospect of estimating the long term cost-effectiveness of these treatments.

**Results:**

Centres consented 5560 patients. Of the 4240 patients who had been in the study for a least one year, annual review data were available for 3730 (88.0%). Of the patients who had been in the study for at least two years and three years, subsequent annual review data were available for 2055 (78.5%) and 265 (71.8%) patients respectively. Baseline characteristics and a small but statistically significant progression of disease were similar to those reported in previous pivotal studies.

**Conclusion:**

Successful recruitment, follow up and early data analysis suggest that risk sharing schemes should be able to deliver their objectives. However, important issues of analysis, and political and commercial conflicts of interest still need to be addressed.

## Background

Risk sharing schemes represent an innovative and important approach to the problems of rationing and achieving cost-effectiveness in high cost or controversial health interventions. Where such interventions have been shown to be effective, and randomised controlled trials (RCTs) are no longer acceptable, careful use of finite resources demands that health services should pay in proportion to benefit. The key feature of risk sharing schemes is to recognise that, for any intervention, price (and therefore cost to the provider) may be variable whereas effectiveness is fixed. Therefore, whatever cost-effectiveness threshold we choose, we should identify the maximum price a health service is prepared to pay for any given intervention. Whilst this holds in theory, there has been little experience of implementing these schemes in practice, until the Multiple Sclerosis (MS) Risk Sharing Scheme.

The use of Beta-interferon and Glatiramer acetate for multiple sclerosis has been highly controversial, with claims of "postcode prescribing" in the UK. A National Institute for Clinical Excellence (NICE) appraisal of the use of three beta-interferon products and glatiramer acetate, published in January 2002 [1], concluded that they should not be funded through the National Health Service (NHS), as the cost per quality adjusted life year (QALY), estimated by the use of a cost-effectiveness model developed in ScHARR [2], was too high. In the face of considerable opposition from patient and professional organisations and pharmaceutical companies, NICE recommended that the Department of Health and the four pharmaceutical companies involved in manufacturing the drugs should find a way to make them available on the NHS in a cost-effective manner. This led to the MS Risk Sharing Scheme [3], in which the drugs were funded on condition that their effect on disease progression was monitored in a cohort of patients for ten years. Depending on the results observed, potential adjustments to the price of the drugs would be made at intervals to achieve an agreed cost per QALY of no more than £36,000.

We report our experience of undertaking the monitoring study for the initial phase of this innovative scheme, the practical, scientific and political challenges encountered, and lessons for the use of risk sharing schemes for other high cost interventions.

## Methods

### Recruitment

Prescribing Disease Modifying Therapies (DMTs) to patients under the MS Risk Sharing Scheme was permitted from May 2002 in specialist MS centres. The DMTs included in the scheme were three beta-interferon products (Avonex, Betaferon and Rebif in two doses) and glatiramer acetate (Copaxone). Choice of drug was made between clinicians and patients according to usual clinical practice. Recruitment of the centres to the monitoring study began in August 2002 in centres across the UK.

Eligible patients were those over the age of 18 years prescribed DMTs according to Association of British Neurologists (ABN) guidelines [4], for whom an assessment of disability had been made prior to treatment. These were recruited to the study by the clinical teams in the centres. Recruitment formally closed at the end of April 2005 though a small number of participants already in the system were consented to the study after that time. Patients are being followed up annually for ten years, whether or not they switch or discontinue DMT treatment.

### Data Collection

Data were collected at baseline assessment and annual review, and included patient demographics, DMT prescribed and treatment start date, number of relapses, disease duration, and type of MS. The principal outcome measure was the Expanded Disability Status Scale (EDSS) [5], a widely used scale for assessment of disability in MS, employing a range of scores from 0 (normal neurological examination) to 10 (death from MS). Data were recorded on data collection forms and entered onto a study database held at each centre. Anonymised data were exported electronically to the University of Sheffield at regular intervals.

### Data Quality

Study fieldworkers checked the completeness and accuracy of the data recorded in data collection forms on regular centre visits, and resolved queries with clinical staff. Once regular data export was achieved, data validation reports were produced centrally and sent to the fieldworkers for resolution with the centres. These reports were based on acceptable value ranges identified with the clinical co-ordinators. Data source verification of study data against patient notes was undertaken for 446 patients in 63 centres. EDSS assessment was undertaken as far as possible by the same neurologist each year. A study to measure inter-rater reliability of EDSS assessments scores was also undertaken. 138 assessments were undertaken on 69 patients from 36 of the participating centres, with two clinicians reviewing each patient.

### Ethics

Approval for the study was obtained from South East Multi-Centre Research Ethics Committee and research governance approval was obtained from all participating centres.

### Analysis

Analyses presented in this paper are on data exported up to 31^st ^January 2006, from patients starting DMTs since May 2002.

### Descriptive analyses

Simple descriptive analyses of frequency and distribution of data items at baseline and each annual review were undertaken.

### Changes over time

Between baseline and each annual review we compared:

• the proportion of patients switching and stopping DMTs

• the proportion of patients with secondary progressive MS

• annual relapse rates.

We also measured disease progresion by the average change in EDSS score per year between baseline and final review for each patient, and examined the distribution of the changes in sub-groups of patients.

Tests were undertaken for heterogeneity in progression in EDSS scores between subgroups defined on characteristics identified in the literature and in the natural history cohort to be prognostic factors i.e. age at baseline (as a proxy for age at onset), sex, relapse rate and rate of early progression, prior to DMT [6]. Early progression rate was estimated as the mean annual change in EDSS score between reported date of symptom onset and baseline, assuming an EDSS score of zero at symptom onset.

## Results

### Recruitment

71 centres agreed to participate in the Risk Sharing Scheme, and reported prescribing DMTs to 6577 patients between 1st May 2002 and close of recruitment to the study on 30th April 2005. 5560 (85%) of these patients were consented to the monitoring study by 70 of the centres. Excluding 277 patients withdrawn from the study, data on 4966 were exported electronically and were available for analysis. The majority (72%) of withdrawals were because they were found, after consent, not to have met the eligibility criteria. Data were excluded for the further 79 patients who started DMT prior to May 2002 and the 16 patients for whom information about start date was not recorded. The remaining 4,871 patients are included in this analysis, with follow up ranging from 9 to 44 months.

### Data Quality

6050 annual reviews were included in the analysis. Of the 4240 patients who had been in the study for a least one year, annual review data were available for 3730 (88.0%). Of the patients who had been in the study for at least two years and three years, subsequent annual review data were available for 2055 (78.5%) and 265 (71.8%) patients respectively. Data were missing either because the assessments had not been done or the data had not been received at the co-ordinating centre at the time of analysis. Analyses of the baseline characteristics of patients with and without annual review were similar, suggesting that data could be treated as missing at random. 79.4% of first annual reviews were undertaken by the clinician making the baseline assessment. The inter-rater reliability exercise produced Kappa scores of 0.59 (95% CI 0.51, 0.66), 0.71 (95% CI 0.63, 0.79) and 0.85 (95% CI 0.77, 0.93) for full agreement and agreement to within 0.5 and 1.0 EDSS score respectively.

For key data items (age, sex, DMT, MS type, EDSS score, relapses), rates of missing data ranged from 0.01% to 4.9%. Data source verification identified no inconsistencies between data collection forms and patient clinical records in recorded DMT, four in EDSS score, and 17 (6.9%) in number of relapses.

### Baseline characteristics of the participants

**Figure 1:**
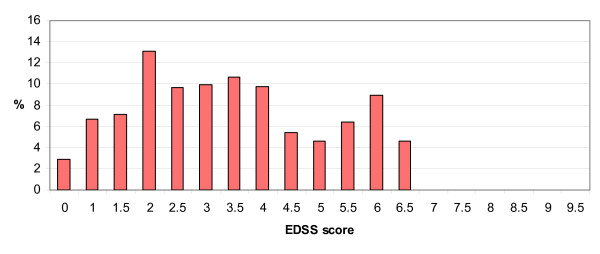
**Distribution of EDSS scores at baseline**.

Baseline characteristics reflected those reported in other large pivotal studies of patients on DMTs [7]. Women represent three quarters of the study cohort (table 1). The mean age of patients at baseline was 39.3 years, mean duration of disease from symptom onset was 8.5 years, and the mean number of years since diagnosis was 5.2 (table 1). The ABN guidelines restrict prescription of DMTs to patients with two relapsing forms of MS: 85.8% of patients had relapsing remitting (RR) MS, with the remainder having a relapsing form of secondary progressive (SP) MS. The distribution of EDSS scores was bimodal with peaks around EDSS 2 and 6 (fig. 1), and a mean of 3.4, standard deviation (SD) 1.7 (table 1), a pattern seen in other large cohort studies [6]. The mean EDSS score for patients with SPMS was 5.5 (SD 1.1) compared with 3.1 (SD 1.5) for patients with RRMS.

**Table 1:** Baseline characteristics of patients recruited to start DMT for the first time (n = 4871)

Female (%)	3644	(74.9)
RR MS (%)	4176	(85.8)

Age in years, mean (SD)	39.3	(9.1)

Years since diagnosis, mean (SD)	5.2	(5.8)

Years since onset, mean (SD)	8.5	(7.4)

Relapses in prior two years, mean (SD)	2.9	(1.2)

EDSS score, mean (SD)	3.4	(1.7)

DMT (%)		
w	1982	(40.7)
x	857	(17.6)
y	1153	(23.7)
z	878	(18.0)

### DMT use

DMT use reflects clinical practice as determined by patient and doctor choice, ABN guidelines and, particularly in SPMS, licensing regulations. One DMT was prescribed to almost twice as many patients as the other DMTs (table 1). At first annual review 278 of 3730 (7.5%) patients had stopped DMT treatment. The cumulative proportion of stoppers increased to 12.5% (256 of 2055) at second annual review and to 14.0% (37 of 265) at third annual review. The proportion of patients switching between DMTs increased from 3.9% (145 of 3730) at first annual review to 8.2% (169 of 2055) at second and 13.6% (36 of 265) at third, in line with case series [8].

### Changes over time in MS type, disease progression and relapse

For those patients for whom we had data at third annual review, the proportion with SPMS increased from 14% (37 of 265) to 25% (67 of 265) from baseline (table 2).

**Table 2:** MS type, relapse rate and mean EDSS score at each annual review point

	**Patients with AR1 (n = 3730)**		**Patients with AR2 (n = 2055)**		**Patients with AR3 (n = 265)**	
	**Baseline**	**AR1**	**Baseline**	**AR2**	**Baseline**	**AR3**
SP MS (%)	536 (14)	664 (18)	323 (16)	456 (22)	37 (14)	67 (25)

Annual relapse rate, mean (SD)	1.43 (0.56)	0.66 (0.91)	1.48 (0.65)	0.58 (0.88)	1.40 (0.56)	0.46 (0.76)

EDSS score, mean (SD)	3.34 (1.67)	3.45 (1.84)	3.51 (1.69)	3.76 (1.94)	3.59 (1.65)	4.00 (1.97)

The mean number of relapses in the two years prior to baseline assessment was 2.9 (table 1). The annual relapse rate in those patients reaching first annual review reduced from 1.43 at baseline to 0.66 at first annual review (table 2).

In the 3730 patients who had a first annual review, mean edss score increased from 3.34 at baseline to 3.45 at AR1 (table 2). This small increase in EDSS score continued through subsequent ARs with a mean increase of 0.10 to 0.15 per year. The average change per year in EDSS score for RRMS patients was +0.125 (SE 0.016).

**Figure 2:**
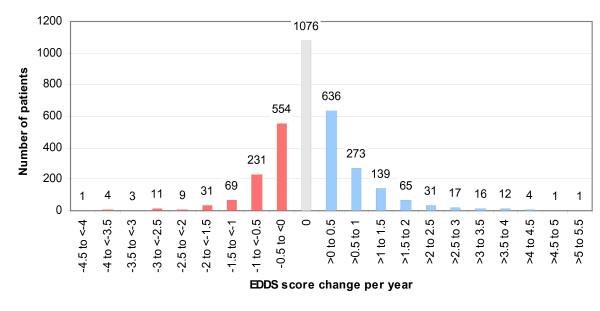
**Distribution of each patient's average annual EDSS score change**.

Although there was an increase in mean EDSS at each annual review, this resulted from a mixture of increases and decreases (progressions and regressions) for individuals. The distribution of the mean change in EDSS score for each patient for the time they were in the study is shown in fig 2. There are almost as many patients with mean overall regression (n = 913) as progession (n = 1195).

### Heterogeneity

The annual EDSS progression rate was one and a half times higher in men than in women, and in those aged 40 years or over than in younger patients, though only the latter was statistically significant (table 3). There was no evidence of any relationship between relapse rate at baseline and EDSS progression. Mean progression rate between ARs was slightly higher in those patients with more rapid progression prior to baseline, though again this finding was not statistically significant.

**Table 3:** Mean annual EDSS score change by potential prognostic factors

		**Mean**	**N**	**Std. Error of Mean**	**Test**	**P**
**Sex**	**Male**	0.169	783	0.031	Mann-Whitney	0.204
	**Female**	0.111	2399	0.018		

**Age at baseline**	**0–39**	0.102	1803	0.021	Mann-Whitney	0.014
	**40+**	0.155	1381	0.023		

**Relapses in two years prior to baseline**	**2**	0.146	1480	0.023	Kruskal-Wallis	0.878
	**3**	0.099	954	0.029		
	**4+**	0.116	750	0.032		

**Progression rate prior to baseline (>2 reviews)**	**0 to 0.464**	0.169	775	0.033	Mann-Whitney	0.455
	**>0.464**	0.213	806	0.035		

## Discussion

We have demonstrated that it is feasible to meet the practical challenges of undertaking a risk sharing scheme within the NHS. This also illustrates the potential of the NHS as a 'population laboratory' and further supports the development of research networks [9]. We have collected baseline and annual review data for up to three years on over 5000 patients recruited from 70 centres across the UK, and continued collection of these data for the remaining seven years of the scheme should allow identification of a price at which the NHS can provide DMTs in a cost-effective manner. This is important because the debate about the cost-effectiveness of DMTs continues, despite the literature on their effectiveness in certain patients, and their wide international use [8]. We are not aware of any other published evidence showing the practicality of implementing risk sharing schemes.

The MS Risk Sharimg Scheme Monitoring Study has several advantages for measuring cost-effectiveness in usual clinical practice. Because our cohort includes the majority of patients prescribed DMTs over the recruitment period in the vast majority of prescribing centres, and since we have not altered usual clinical practice in these centres, the external validity of our study is very high. This cohort of patients represents the vast majority of those prescribed DMTs according to ABN guidelines over the recruitment period, and the data collected show the expected demographic characteristics in terms of age, sex and MS type. The DMT prescribing pattern in the cohort reflects clinical practice in the UK, though the considerable variation found between centres requires further investigation. The study already has follow up as long as any published RCT and will be the largest cohort of DMT treated patients with long term follow up. The ScHARR MS model predicts long term EDSS progression under DMT treatment using natural history data from a cohort of Canadian MS patients followed up for 25 years, together with a reanalysis of RCT data on the impact of treatments on progression [2,6]. There are no concurrent control patients, and it has been argued that a long term RCT would have been more appropriate than the MS Risk Sharing Scheme [10]. However, it would have been unacceptable to many patients and clinicians to randomise to no treatment (since benefit on relapse rates has already been proven). Randomising between DMT products was not acceptable to the pharmaceutical companies who were parties to the Risk Sharing Scheme. The study will also enable accurate estimation of rates of switching and stopping DMTs as well as the costs and quality of life associated with different EDSS states which it is essential to know to accurately estimate the cost-effectiveness of the DMTs. We have ensured a complete transmission of all entered data at the time of the analysis reported here, locking the database on 31st January 2006. In these data between 12.0% (for those in the study at least one year) and 28.2% (for those in the study at least 3 years) of interim EDSS review scores were missing. However, the cost-effectiveness analysis linked to pricing, proposed in the original Health Service Circular [3], will not use these interim scores. The analysis depends only on the baseline score and the last confirmed score collected before analysis. Recording final review scores will therefore be a priority for future data collection. Cost-effectiveness may differ between subgroups of patients at different stages of the disease and subgroup analyses will be important to address this.

Over the first three years, we have found small but statistically significant progression of disease, as measured by mean EDSS changes, in MS patients on DMTs. This is consistent with previous, short term, RCTs where the small changes observed have made it difficult to assess the effect of DMTs on disease progression [7]. However, the small changes in mean EDSS between baseline and each annual review mask considerable variation in individual patients. At annual reviews EDSS scores have decreased almost as often as increased, compared to baseline. This variation could be due to a number of factors. With EDSS, as with any outcome measure, there is some intra- and inter-observer variation, though when we restricted our analysis to patients whose EDSS assessment was made by the same clinician at baseline and annual review, the findings were unaffected. It is also possible that the patients whose EDSS scores have decreased were still recovering from relapse at the time of baseline assessment. A third possibility is that the lower EDSS scores represent true (if temporary) regressions in disability in these patients. Without a control group, it is hard to say whether this level of regression is due to the effect of DMTs, or whether it occurs in the disease's natural history, though by the end of the ten year follow up, there will be a much clearer picture of overall progression on these drugs. The annual relapse rate at first annual review was reduced by 54% compared to baseline. However, differences in the estimation of relapse rate at baseline and at annual review and the possibility of a regression to the mean caused by patients with temporarily high relapse rates being entered into the scheme mean that caution is required in interpreting this finding. Nevertheless, the reduction observed is in line with that seen in previous RCTs [7]. The results from data source verification, the inter-rater reliability study and low rates of missing data are supportive of data quality, as is the EDSS distribution pattern, which is as would be expected from previous cohort studies [6].

Our data indicate possible heterogeneity in disease progression in specific population subgroups. In particular, older age at disease onset may be associated with faster rate of progression in disability. This heterogeneity is consistent with other recently published reports [11,12], and has implications for the future planned analyses of the data to determine potential price adjustments. The natural history cohort used in the analysis should be adjusted from the Canadian study population to match the characteristics of patients entering the Risk Sharing Scheme. It is also important that the natural history data is validated, since there is evidence that the incidence of MS is changing and that the natural history may be becoming more benign [11,13]. To ensure a level playing field in the assessment of cost-effectiveness of the different DMTs, case-mix adjustment will be necessary. Further complexity is added by patients switching and stopping drugs, and this needs to be addressed in the analysis. Additionally, the cost effectiveness may depend on characteristics that influence responsiveness such as neutralising antibodies and relapse frequency.

We have shown that the practical difficulties in establishing the Risk Sharing Scheme can be overcome, and the scientific challenges we have identified can be addressed through appropriate methods of analysis. However, perhaps the most important threat to the NHS's ability to determine the cost-effectiveness of DMTs comes from the tensions inherent in such a scheme. These arise from the differing interests of the Department of Health, the pharmaceutical companies, researchers and patients. The commercial risks associated with the scheme led to the involvement of the companies as well as the Department of Health in the scheme's governance. The scheme was retendered but the ScHARR consortium decided not to apply as they were not happy with the proposed arrangements for data access and publication rights, and the scheme is now being undertaken by a contract research organisation. The data that is being collected by the contract research organisation for use in future publications may not be the same as the data we have presented here. The data from the scheme is owned by the MS Trust.

## Conclusion

Whilst it has been possible to meet the operational challenges in delivering the MS Risk Sharing Scheme, important scientific challenges and conflicts of interest remain in this highly controversial area. Risk sharing schemes represent an important way for health services to ensure cost-effective provision of high cost health care interventions, so long as the research is independent of the parties to the schemes.

## Competing interests

KA has received research funding from Schering Health Care Limited to undertake research in reproductive healthcare, and has acted as a paid consultant to consultancy companies who undertake work for the healthcare industry generally; MB and JP have been given financial support by Biogen Limited, Schering Health Care Limited, Serono Limited and Teva Pharmaceuticals Limited/Aventis Pharma to attend scientific symposia, give lectures and (for JP) to participate in drug study investigations; PT has acted as a paid consultant on behalf of Biogen Limited on the treatment of multiple sclerosis with natalizumab.

All other authors declare that the answer to the questions on your competing interest form are all "no" and therefore have nothing to declare.

## Authors' contributions

Study concept and design: JN, MB, JP, AOH, KRA, JBC, GE

Acquisition of data: CLC, MP, TC, MB, JP, JN

Analysis and interpretation of data: JN, TC, MP, CLC MB, JP, AOH, KRA, JBC, PT, NJC, GE

Drafting of the manuscript: MP, CLC

Critical revision of manuscript: CLC, MP, MB, JP, TC, AOH, KRA, NJC, GE, JBC, PT, JN. JN is the guarantor for the paper.

## Pre-publication history

The pre-publication history for this paper can be accessed here:


